# Supplementary material for: Complex Sound Discrimination in Zebrafish: Auditory Learning Within a Novel “Go/Go” Decision-Making Paradigm
Source: Animals (Basel). 2025 Nov 29;15(23):3452. doi: 10.3390/ani15233452 (PMC12691207; doi:10.3390/ani15233452)
Supplement: Supplementary file 1 [file animals-15-03452-s001.zip › animals-3849064-supplementary.pdf]

**Supplementary material**

## Section S1: Speaker calibration using the DolphinEar DE-200 hydrophone

The DE200 is an omnidirectional hydrophone with an overall frequency-response bandwidth of 1 Hz-24000 Hz. The internal amplifier/earphone system limits the frequency response to the normal human hearing range. We used the following protocol to obtain a frequency-response curve using the DE-200 hydrophone for our Bluetooth speaker placed below the test tank.

*Signal design, equipment setup and data acquisition*

- We located the hydrophone in the center of the tank, away from any surfaces (including the water surface and tank walls) to avoid interference from reflected sound.
- To minimize reflections, we used burst signals to minimize reverberation interference. We used 100 ms tone pips with a 10 ms rise and fall time generated using Audacity. All tones had the same peak-to-peak voltage of 1 volt. Frequencies tested ranged from 200 Hz to 3 kHz with 200 Hz increments. Signals were high-pass filtered above 200 Hz to eliminate any low frequency noise.
- We connected the hydrophone to the DolphinEar amplifier (with an input impedance >100k ohms) and the accompanying sound acquisition software. We ensured all connections were watertight and secure and powered the hydrophone via its dedicated power supply.
- The hydrophone was connected to a miniPC desktop, and the acoustic response was recorded using the DolphinEar SPEC software. A power spectrum of the recorded sound was potted using Audacity software.
- Amplifier gain was checked to be consistent across the frequency range of sounds to be tested. We adjusted gain to an appropriate level.

*Note:* A calibration sheet for the DE200 was not available, so a gain using V/μPa was not calculated. When available, the measured voltage can be converted to sound pressure using the following formula to determine the SPL:

SPL = (VoutdB - G) + OCV in dB/1μPa.

The frequency-response function for this range was relatively flat with ±3 dB. Frequencies were tested between 200 Hz and 3 kHz. There was a steep roll off below 400 Hz.

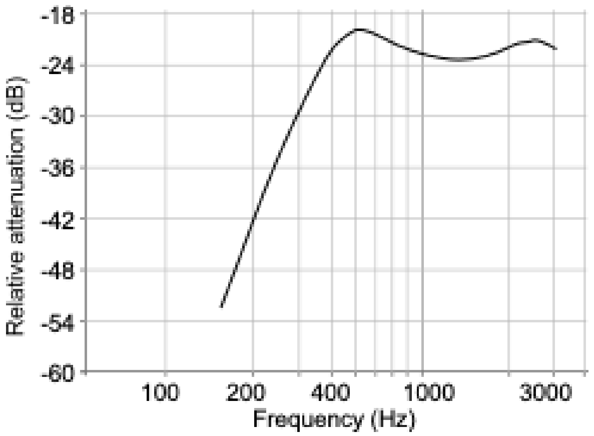


**Supplementary Figure S1.** Frequency-response curves for speaker calibration under water. Frequencies are plotted on a log scale.

**Section S2: Methodology Development and Analysis**

*S2.1. Exploratory swim patterns*

We first tracked the normal free-swimming behavior of fish in the test tank environment to ensure that they did not stay solely at one end (Fig. S2). Exploratory swim patterns and preferred locations varied among fish, but by and large they tended to explore the entire tank space. Frequently, fish also tended to swim in proximity (within 20 to 40 mm) of the backlit screen display. Data shown are only for the first 30 seconds of swimming for clarity of tracking although the initial baseline activity before initiating training and test trials (b1) and after completion of all testing (final baseline interval, b2) are shown. Each baseline duration was six minutes.

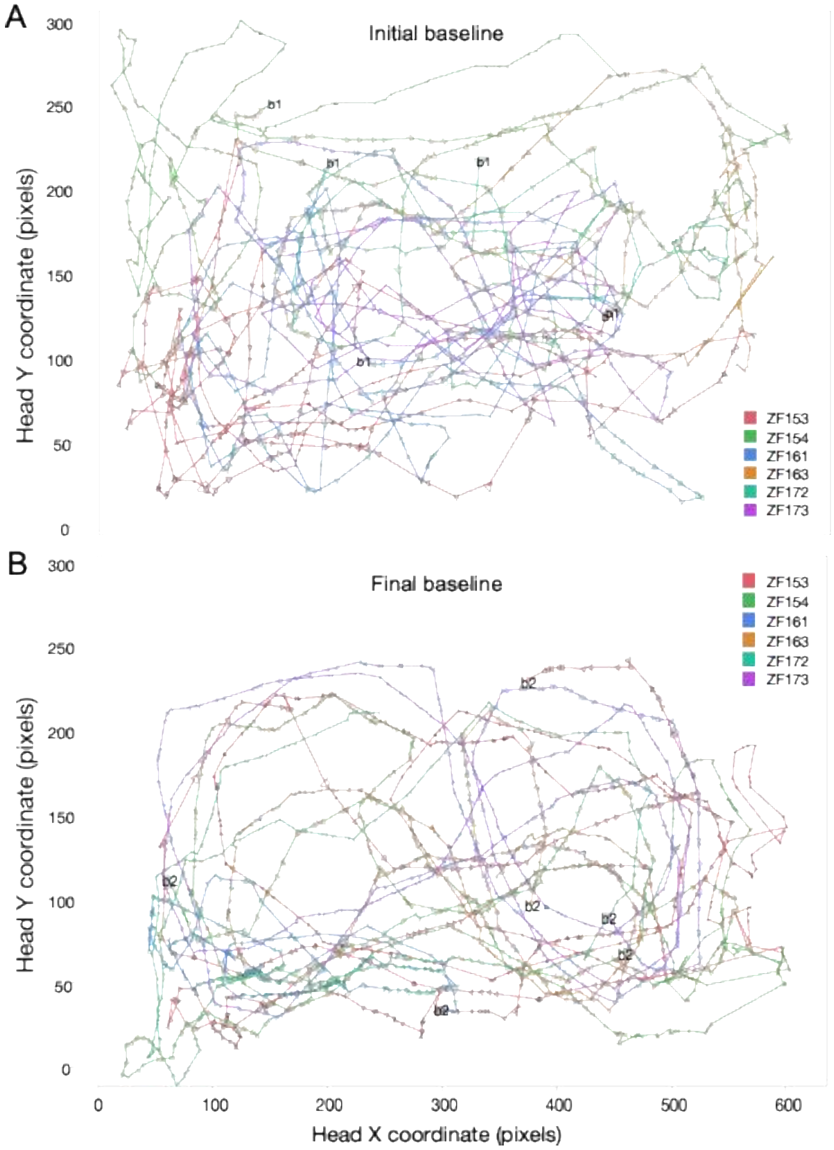


**Supplementary Figure S2.** Timeline bubbleplots with trails to show the track of each animal's color-coded swim trajectory during the initial (A) and final (B) baseline interval. Tracks with small triangular markers per video frame are superimposed from the same six animals. Triangular marker size is reduced to accentuate the track traces. Trace ends, labeled as the "b1" and "b2" indicate the terminal position at the end of the same-colored

track for the initial and final baseline condition, respectively. A larger space between triangles translates to a higher swim speed. The LCD screen was present on the right at X-value of ~600.

*S2.2. Single-sound conditioning profiles and analysis*

We first used a unidirectional "Go" paradigm to train fish to predict and respond to a single sound cue. Here, a single sound stimulus block of eight trials where the rewarding unconditioned stimulus (US) was paired with a CF- and the aversive or fearful US with an FM-type of sound in different animals. Location data from single stimulus presentations in six zebrafish, shown as density plots in Figure S3A, illustrate the "centers-of-gravity" of fish locations during the gap interval after eight trials of training. On average, fish moved away from the screen in response to the DFM sound cue and towards the screen-side of the tank after the NCF sound cue. These data established "distance from screen" as a useful behavioral measure for discrimination between the two sound types.

To illustrate typical learning dynamics across trials, we show a scatterplot of fish locations during each trial overlaid with a spline plot for the single-sound learning condition across all eight trials in individual fish (Fig. S3B.) Both fish showed a general screen-side bias. Regardless, each fish exhibited an increasing tendency to predict the US from the sound and swim either towards or farther away from the screen. These plots show that place preference exhibited in response to a sound is not a reflexive response but gradually learned across runs so that during successive gap intervals, a fish positions itself at an appropriate location prior to the onset of video playback for the US. In short, fish predict the US from the sound and learn to swim either towards or away from the screen over successive runs. In follow-up sessions, this combination was reversed (NCF-fear, DFM-reward) to rule out intrinsic bias of a sound type for its association with the US. These single sound sessions provided proof of concept for perception of complex sounds and sound conditioning, but not necessarily discrimination between the two sounds.

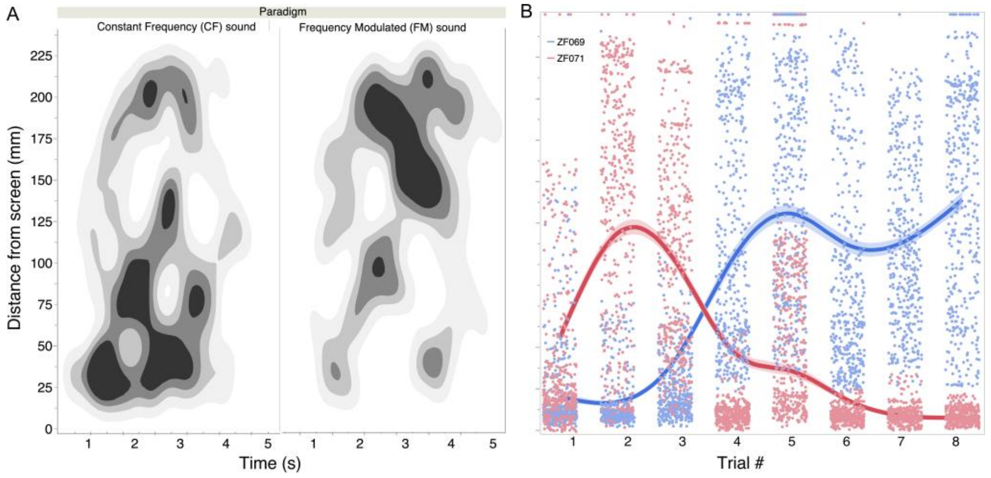


**Supplementary Figure S3**. A. Density plots of average locations (n = 6) across all runs during the 3-s duration between the end of the auditory stimulus (NCF on the left and DFM on the right) and playback of the video clip on the display screen. B. Scatterplots with splines showing learning over time in two individuals that were presented with either a NCF$_{rew}$ (red: reward) or a DFM$_{fear}$ (blue: fear) sound over 8 trials.

Encouraged by these observations and for more extensive testing we switched to dual stimulus conditioning. Each animal was conditioned with both sounds in a

randomly selected manner in succession within a single, continuous experimental session. These data did not show clear differences, however, between distance from screen measure for the two conditions. Rather, the random presentation of each CS-US pair appeared to confuse the fish during early stages of conditioning. This was not surprising given the dynamic nature of exploration that includes frequent turning in free-swimming zebrafish.

Rapid context switching by randomly alternating presentation of the two sounds to naïve fish in the beginning can be a pitfall in this version of associative conditioning. We discovered this can lead to a lingering effect of the preceding context. This prompted us to first understand the intra-trial and inter-trial sources of potential bias in the process of learning naturally. Knowing these biases can be helpful in both arriving at an optimal training paradigm and data analysis strategy.

*S2.3. Context dependence of conditioned place preference*

Initial analysis on a subset of fish during the development phase of the assay involved calculation of a Place Preference Index (PPI) using the following equation.

$$\text{PPI} = \frac{\boldsymbol{screen-side\ preference\ (s)}}{\boldsymbol{screen-side\ preference\ (s) + far-side\ preference\ (s)}}$$

where PPI is a ratio of the time spent on the screen-side of the tank to the time spent on both ends of the tank. PPI values ranged from 0 to 1. The tank was divided into 3 zones of equal length and the end zones were designated as the screen-side and the far-side (away from the screen). The middle zone was considered neutral. Screen- and far-side preferences were calculated by counting the number of frames in which the fish was located on either side of the tank from the ZebraZoom tracking. A sum of the time difference between successive frames recorded during the gap interval was used to estimate the time spent within that zone/side.

Variability in swim patterns resulting from frequent turns can be an intrinsic swim strategy to evade a predator but also result from a level of confusion due to switching context, i.e., exposure to reward vs. fear associations across the two blocks within our training paradigm. Therefore, for a deeper understanding of the possibility of context-bias from exposure to a preceding CS-US pair presented in Block 1 (either NCF-reward or DFM-fear) with the alternate stimulus in Block 2, we used ZebraZoom-extracted x-y data to calculate the PPI for each trial during successive exposures to the CS. We used the two-block dataset to test for a possible effect of contextual bias in the learning process (Fig. S4A). Here, the NCF-reward ($NCF_{rew}$) association was presented prior to the DFM-aversion or fear ($DFM_{fear}$) consecutive trials in six fish and *vice-versa* in another six fish.

In the opposite context, the first block consisted of $DFM_{fear}$ conditioning followed by a second block of $NCF_{rew}$ conditioning (Fig. S4B). Here, fish exposed first to the set of FM-fear conditioning trials exhibited a decrease in PPI. By the 5th and 6th trial of this block of aversive training, four out of six fish had decreases in PPI (decreased preference for the screen-side of the tank). In summary, when the $NCF_{rew}$ pair followed aversive training, the reward was not as effective in eliciting the expected positive shift in the PPI. From these analyses, we discovered that the learning process can be 'contaminated' by the previous context, e.g. a generalized lingering fear of a sound. The $DFM_{fear}$ conditioning during Block 1 can also influence $NCF_{rew}$ conditioning during the first one or two runs of Block 2.

*S2.4. Sound-directed prediction and inter-trial response dynamics*

We presented a total of 12 stimuli in two successive blocks of 6 repeat trials (either CF or FM). Fish with a relatively low PPI during the auditory to visual (CS to US) gap interval indicated that they moved away from the screen at the onset of

the fear stimulus. We used hierarchical clustering to group fish based on the sequential values of PPI (shown as heatmaps in Fig. S4A). Four out of 6 fish trained with the first block as the $NCF_{rew}$ pair (set of six trials) exhibited a general increase in PPI (successively lighter shades of blue or darker shades of red) over the course of Block 1. Specifically, their preference for being nearer to the screen-side of the tank increased in the last two trials of the first block and then decreased during the $NCF_{rew}$ conditioning during the next 6 trials in Block 2 (Fig. S4A). We were also curious whether the test animal is primed or "biased" to swim in a particular direction during the onset of $NCF_{rew}$ after the $DFM_{fear}$ conditioning trial (Fig. S4B).

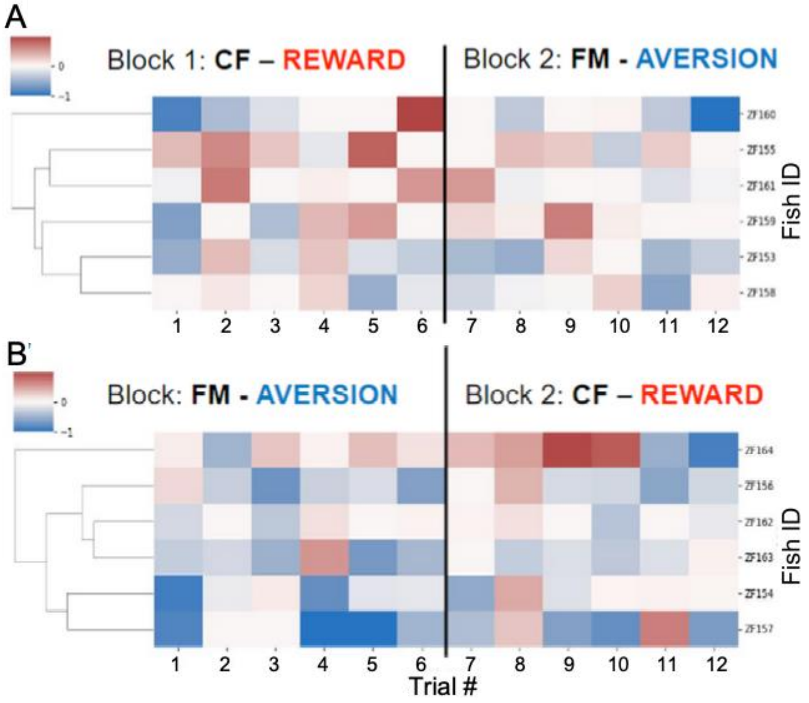


**Supplementary Figure S4.** Heat maps showing intra-trial positioning dynamics and across-trial hysteresis. Progression of shifts in PPI (ΔPPI) during sound discrimination sessions where both CS-US pairs (NCF-reward/DFM-fear) were presented.to each animal during twelve successive trials within the same session. Top panel shows conditioning with the $NCF_{rew}$ sound presented in Block 1 and lower panel with the $DFM_{fear}$ sound presented in Block 2. Dendrograms depict clusters of individuals based on proximity of the pattern of PPI progression during dual-stimulus conditioning. Darker shades of red on heatmaps indicate an increase in value of PPI and darker shades of blue indicate a decrease in the value of PPI within each trial.

*S2.5. Hysteresis effects*

To estimate the state of the animal at the beginning of successive trials within the same training session, we computed the correlation between an animal's initial response to the CS and a change in its pre- vs. post-conditioning PPI. We measured the shift in PPI over the first 0.3 s of the gap interval to test its correlation with the initial distance from screen at video onset. These data are shown as scatterplots in Figure S5A for Block 1 with FM-aversion conditioning, and in Figure S5B for Block 2 with $NCF_{rew}$ conditioning for the corresponding trials in the same six animals. Over the first 3 runs (shown for 6 animals) where the $NCF_{rew}$ conditioning preceded the $DFM_{fear}$ conditioning, the approach response 0.3 s after video onset was positively correlated with a change in PPI during the gap interval in Block 1 (P =

7.7E-5, Bonferroni = 0.01) and in Block 2 (P = 0.04, Bonferroni = 0.05). The reduction in the slope of the regression line shows that the DFM$_{fear}$ conditioning during Block 1 influences NCF$_{rew}$ conditioning during runs 1 to 3 of Block 2. The approach response exhibited a positive slope for regression after the CF (P = 0.035).

During DFM$_{fear}$ conditioning in Block 2, there was a negative correlation between the initial approach towards the screen during the gap interval and a shift in PPI during each conditioning trial (Fig. S5B). The regression line in this plot showed a negative slope after the DFM$_{fear}$ conditioning (P = 0.006). This inversion in the correlation was consistent with our Go/Go training paradigm where the fish must decide between opposite swim directions.

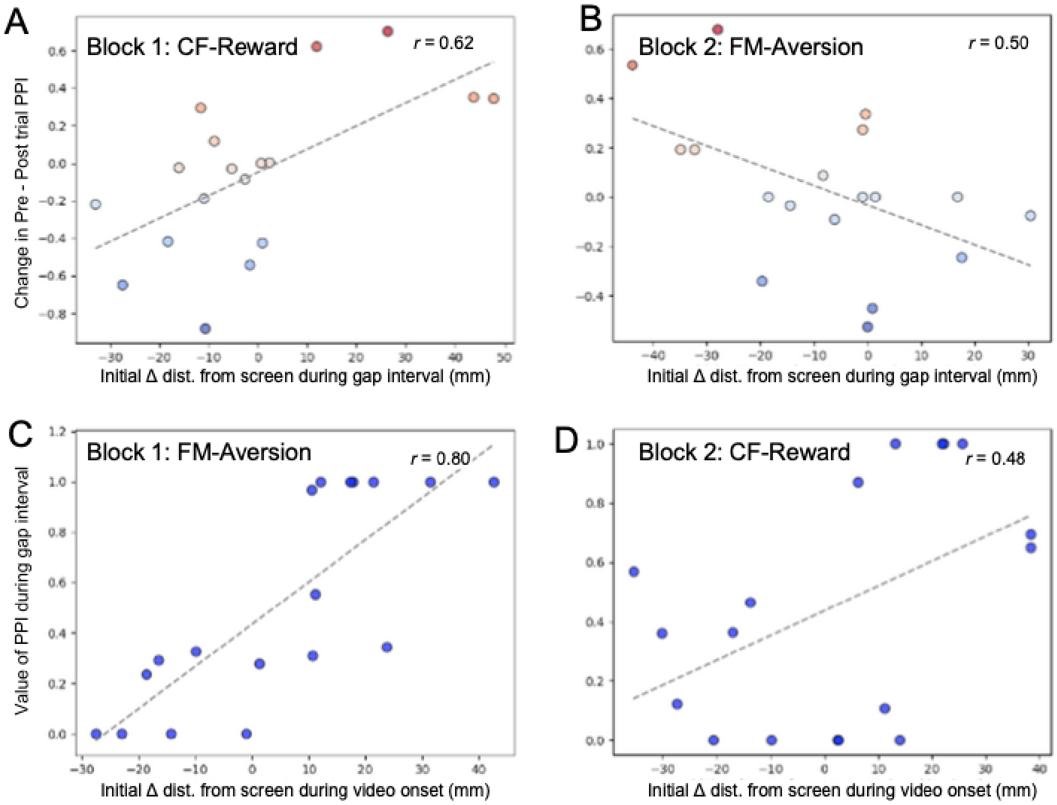


**Supplementary Figure S5.** Scatterplots showing correlation of change in PPI (ΔPPI) and initial response to NCF$_{rew}$ conditioning over runs 1 to 3. B. Scatterplot for correlation between same variables as in A for DFM$_{fear}$ conditioning in Block 2. Graded red colors inside circle symbols are from heatmaps for PPI and indicate an increase in PPI while blue indicates decrease in PPI during each trial. C and D. Relationship of PPI to distance from screen at onset of US (post-CS presentation) showing that DFM$_{fear}$ pair has a greater lingering effect than the NCF$_{rew}$ pair.

*S2.6. Dual-sound discrimination training*

To demonstrate discrimination between two sounds, we needed to train the same fish more effectively to the presentation of both sounds within a single training session. For dual-sound training, we therefore conditioned fish to alternating blocks of NCF$_{rew}$ and DFM$_{fear}$ trials – one block for each CS-US pair. Pilot data from the first four animals tested are presented as density plots for the final run (run # 16) in Block 2 (Fig. S6).

Averaged density plots show that, on average, fish tend to spend more time near the screen in response to the NCF$_{rew}$ and away from the screen in response to

the DFMfear stimulus (Fig. S6A). This difference was acquired over successive trials so that by the final trial for each CS-US pair, fish were moving in opposite directions, depending on the auditory context (Fig. S6B). The data show that fish learn to distinguish between the two sounds and respond robustly by swimming in opposite directions by the eighth run. In the final version of the Go/Go assay, we limited each of three blocks consisted to six runs each since a directional preference was already present during run #6. The same CS-US pair was repeatedly presented for conditioning purposes in the first two blocks whereas in the third block, CS-US pairs were randomly alternated for testing sound discrimination.

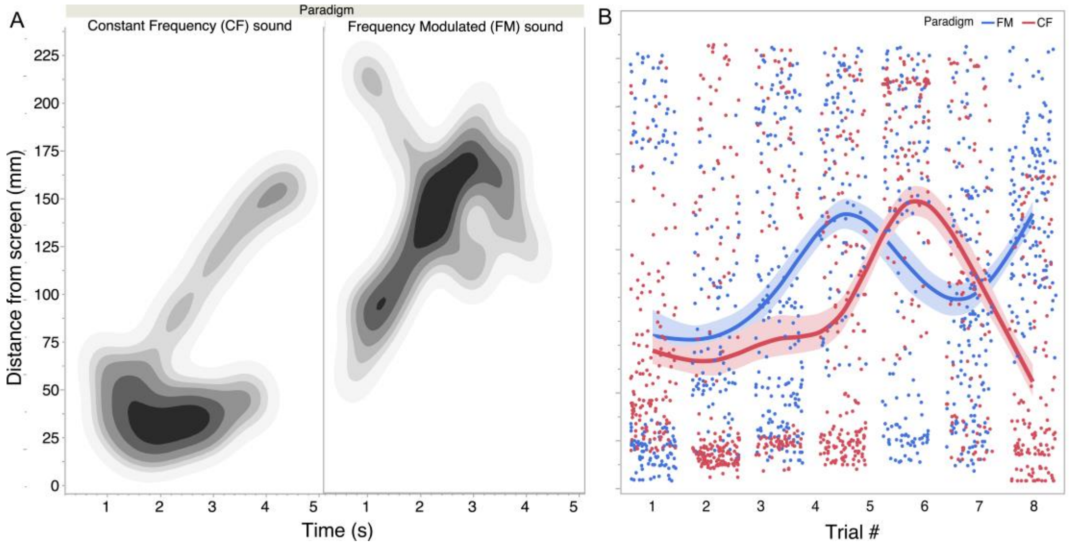


**Supplementary Figure S6**: A. Density plots during final trial of dual sound training in 4 individuals. The plot on the left is in response to NCF*rew* conditioning and on the right shows the predominant location and a moving away trajectory in response to FM-aversion conditioning. B. Scatterplots with splines showing conditioning over time in two individuals that were conditioned to either the NCF*rew* (red) or a DFMfear pair (blue) over 8 trials.

*S2.7. Fish location during sound presentation*

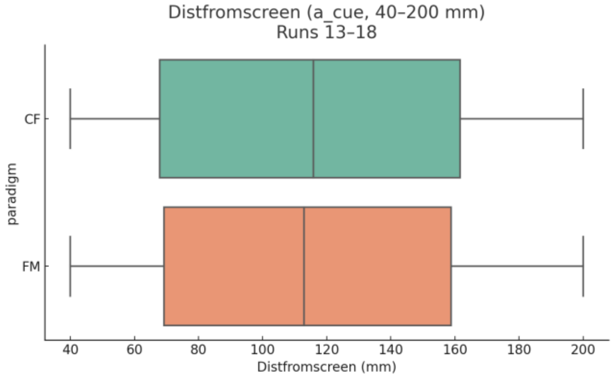


**Supplementary Figure S7.** Box plots to show that there is no difference in the distance from screen measure for fish locations during the 6 s long presentation of the CF and FM sound types. Fish locations were captured for runs 13 to 18 at distance from screen range of 40 to 200 mm to eliminate any effect of screen side-bias.

*S2.8. Memory consolidation evidence*

Within-subject paired comparisons are well powered even with small *n* when effects are consistent across individuals. We observed a reliable shift in state-space structure between Day 1 and Day 2 for each animal, indicating a strong and consistent directional shift across fish as illustrated in the density measures across the two days (Fig. S8).

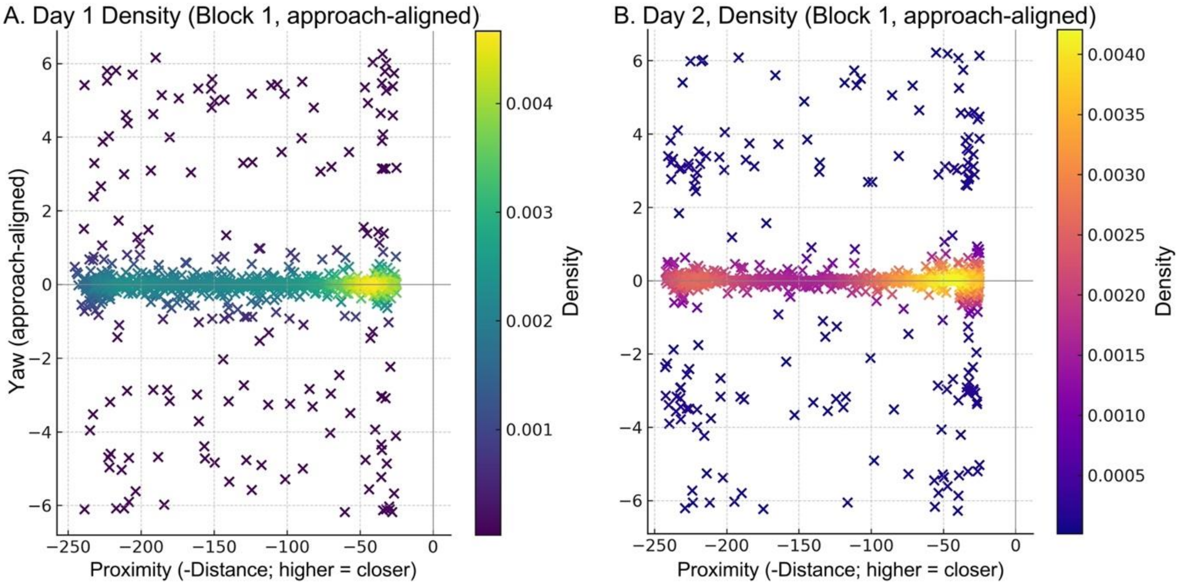


**Supplementary Figure S8**. state-space plots for Day 1 (A) and Day 2 (B) exhibiting qualitative compression of state occupancy. Day 1 reflects an in-progress behavioral state shift rather than a fully stabilized learned response.

## Section S3: Software and media

*S3.1. Experiment software design*

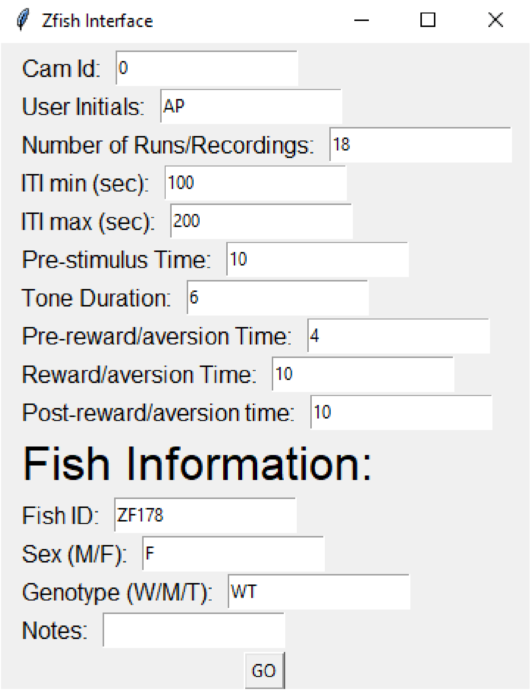


**Supplementary Figure S9**. Custom-software interface for running the Go/Go assay on a desktop computer (Windows 11 OS).

*S3.2. NCF example video (ZF166, 9/26/2024, R5); Filename: ZFishCFresp.mp4.*

https://www.ebi.ac.uk/biostudies/studies/S-BSST2312 DOI: 10.6019/S-BSST2312

*S3.3. DFM example video (ZF166, 9/26/2024, R12); Filename: ZFishFMresp.mp4.*

https://www.ebi.ac.uk/biostudies/studies/S-BSST2312 DOI: 10.6019/S-BSST2312
